# Supplementary material for: Structures of nucleotide-bound Redondovirus Rep protein link conformation and function
Source: PLoS Pathog. 2026 Mar 4;22(3):e1013997. doi: 10.1371/journal.ppat.1013997 (PMC12981564; doi:10.1371/journal.ppat.1013997)
Supplement: S1 Table — (PDF) [file ppat.1013997.s001.pdf]

Supplemental Table 1. Crystallographic data collection and refinement statistics for fbRep-ED 2-114.

PDB	9PQF
Data Collection	
X-ray Source	NSLS-II, Beamline 17-ID-1 (AMX)
Wavelength (Å)	0.92
Temperature (K)	100
Detector	DECTRIS EIGER X 9M
Resolution range (Å)	45.13 - 1.8 (1.83 - 1.8)
Space group	I 2 2 2
Unit cell (a,b,c,α,β,γ)	50.944 62.974 64.705 90 90 90
Unit Cell Volume (Å^3)	207585.079
Total reflections	74196 (3704)
Unique reflections	9971 (475)
Multiplicity	7.4 (7.8)
Completeness (%)	99.98 (100.00)
Mean I/sigma(I)	4.79 (0.98)
Wilson B-factor (Å^2)	23.34
R-merge[b]	0.1938 (0.4328)
R-meas[c]	0.2087 (0.466)
R-pim[d]	0.07636 (0.1707)
$CC_{1/2}$	0.983 (0.816)
CC*	0.996 (0.948)
Model Refinement	
Resolution range (Å)	45.13 - 1.8 (1.83 - 1.8)
Reflections used in refinement	9970 (475)
Reflections used for R-free	982 (54)
R-work	0.1799 (0.2460)
R-free	0.2179 (0.2920)
CC (work)	0.969 (0.886)
CC (free)	0.945 (0.895)
Number of non-hydrogen atoms	961
macromolecules	904
ligands	8
solvent	49
Protein residues	113
RMS (bonds) (Å)	0.007
RMS (angles) (°)	0.90
Ramachandran favored (%)	96.40
Ramachandran allowed (%)	3.60
Ramachandran outliers (%)	0.00
Rotamer outliers (%)	0.98
Clashscore	1.09
Average B-factor (Å^2)	27.91
macromolecules	27.54
ligands	28.89
solvent	34.51
